# Theoretical study of strain-dependent optical absorption in a doped self-assembled InAs/InGaAs/GaAs/AlGaAs quantum dot

**DOI:** 10.3762/bjnano.9.99

**Published:** 2018-04-04

**Authors:** Tarek A Ameen, Hesameddin Ilatikhameneh, Archana Tankasala, Yuling Hsueh, James Charles, Jim Fonseca, Michael Povolotskyi, Jun Oh Kim, Sanjay Krishna, Monica S Allen, Jeffery W Allen, Rajib Rahman, Gerhard Klimeck

**Affiliations:** 1 Network for Computational Nanotechnology, Department of Electrical and Computer Engineering, Purdue University, West Lafayette, IN 47907, USA; 2 Korean Research Institute of Standards and Sciences, Daejeon 34113, South Korea; 3 Department of Electrical and Computer Engineering, Ohio State University, Columbus, OH 43210, USA; 4 Air Force Research Laboratory, Munitions Directorate, Eglin AFB, FL 32542, USA.

**Keywords:** anharmonic atomistic strain model, biaxial strain ratio, configuration interaction, optical absorption, quantum qot filling, self-assembled quantum dots, semi-empirical tight-binding, sp3d5s* with spin–orbit coupling (sp3d5s*_SO)

## Abstract

A detailed theoretical study of the optical absorption in doped self-assembled quantum dots is presented. A rigorous atomistic strain model as well as a sophisticated 20-band tight-binding model are used to ensure accurate prediction of the single particle states in these devices. We also show that for doped quantum dots, many-particle configuration interaction is also critical to accurately capture the optical transitions of the system. The sophisticated models presented in this work reproduce the experimental results for both undoped and doped quantum dot systems. The effects of alloy mole fraction of the strain controlling layer and quantum dot dimensions are discussed. Increasing the mole fraction of the strain controlling layer leads to a lower energy gap and a larger absorption wavelength. Surprisingly, the absorption wavelength is highly sensitive to the changes in the diameter, but almost insensitive to the changes in dot height. This behavior is explained by a detailed sensitivity analysis of different factors affecting the optical transition energy.

## Introduction

Self-assembled quantum dots are employed as light absorbers in many optoelectronic devices, such as quantum-dot infrared photodetectors (QDIPs) [1–2], and intermediate-band solar cells (IBSCs) [3–4]. The optical properties of quantum dots (QDs) can be tuned through shape, dimensions and composition of the dots making them attractive for optoelectronic applications. Moreover, their sensitivity to normally incident light make them advantageous over other nanostructures, such as quantum wells, that are insensitive to normally incident light [2].

The absorption coefficient α(λ) of quantum dots is an important parameter that needs to be precisely designed for the proper operation of these devices. An accurate model for the absorption coefficient α(λ) is therefore crucial in the design and prediction of the device behavior. Therefore, this study aims to fill the gaps in current absorption models, namely the atomistic strain and band structure calculations that are needed for accurate description of the bound states. Moreover, doped devices require evaluation of many-particle configuration interaction (CI) calculations for a proper treatment of the optical transitions. The effects of alloy mole fraction of the strain controlling layer and quantum dot dimensions are also discussed.

Self-assembled quantum dots have around 10% lattice strain [5]. Atomistic strain models like that of Keating [6], or anharmonic models [7] are typically used to determine the relaxed atom positions. The anharmonic strain model has additional strain parameters with anharmonic corrections added to the harmonic model, which improves its accuracy. Without anharmonic corrections, the harmonic potential underestimates the repulsion at smaller bond lengths and also fails to capture the weakening of atomic forces at large atomic separation [8].

The anharmonic strain parameters were originally optimized to obtain correct Grüneisen parameters for accurate phonon dispersion calculations [7]. However, we show that the original parameter set cannot reproduce the experimental optical absorption peaks in quantum dots [9]. Using these parameters to determine strain in quantum wells does not agree with the well-known analytical solution of strain in quantum wells. Optimizing the parameters to obtain correct biaxial strain ratios in quantum wells is shown here to improve the accuracy of quantum dot simulations as compared with experimental measurements. The Hamiltonian is constructed with semi-empirical tight-binding with 20-orbital sp3d5s* basis per atom, including spin–orbit interaction (sp3d5s*_SO) [10]. The absorption coefficient is calculated by employing Fermi’s golden rule.

In the following sections the simulated system and the numerical tools employed in simulations are described, then the theoretical aspects of the problem and optimization of the strain model are discussed. Lastly, the results of the simulation are presented including a sensitivity analysis of the absorption to various quantum dot parameters.

## Multi-Million-Atom Simulation

**Figure 1:**
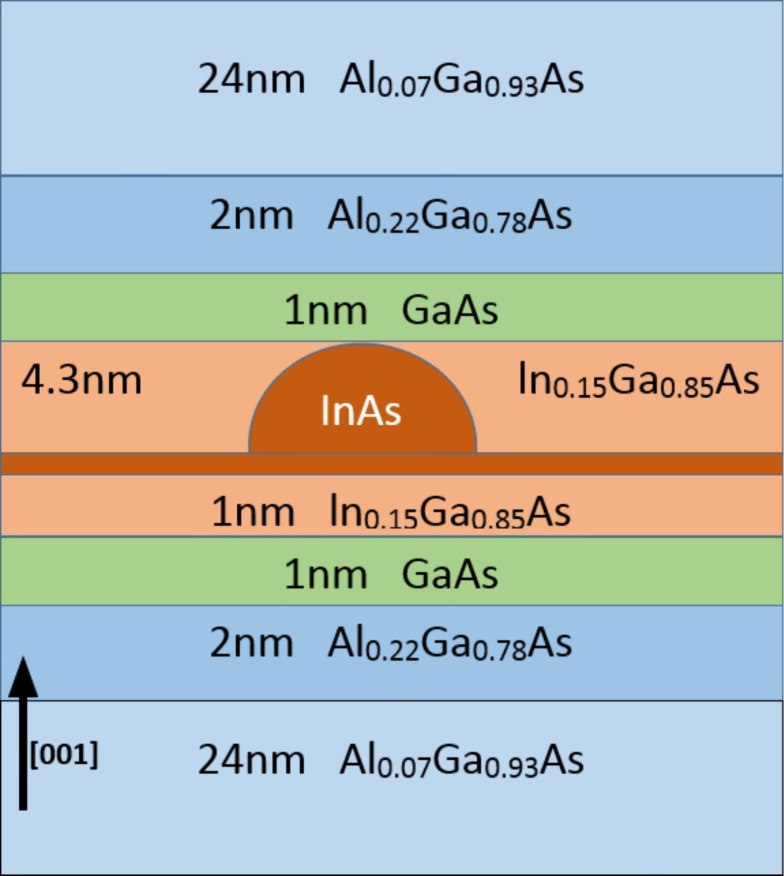
A schematic of the measured and simulated QD system. The dimensions of the simulated structure are 60 nm × 60 nm × 60 nm. The quantum dot is dome-shaped InAs with a base diameter of 20 nm and a height of 5 nm, with a wetting layer of two monolayers. The strain control layer of In_0.15_Ga_0.85_As is sandwiched between two 1 nm layers of GaAs, and two 2 nm layers of Al_0.22_Ga_0.78_As. The rest of the structure is made of Al_0.07_Ga_0.93_As.

**Figure 2:**
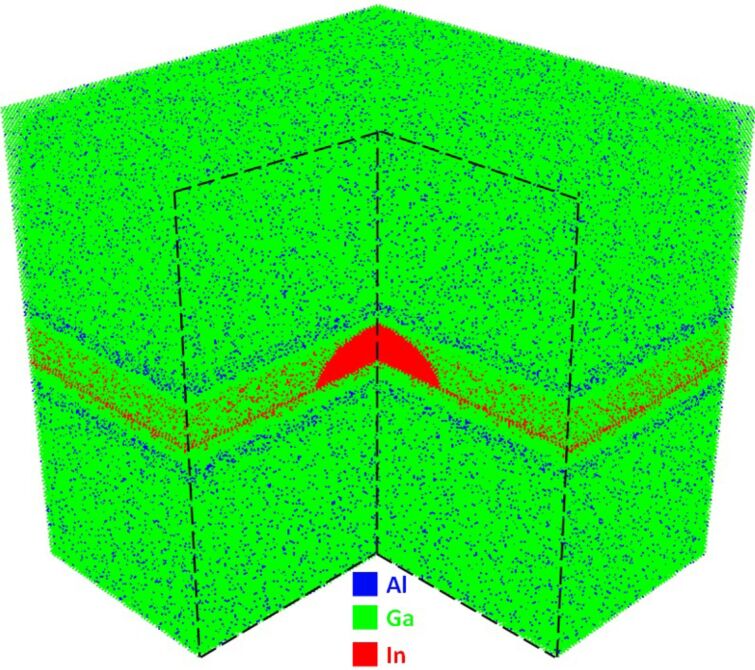
The atomistic grid of the simulated QD showing only cations (Al,Ga,In) to emphasize the randomness of the simulated alloys. The number of atoms used for the strain simulation is approximately 10 million atoms, while for band structure calculations only approximately 1.5 million atoms are used.

As shown in the Figure 1, the investigated QD system [11] has a dome-shaped InAs quantum dot with a base diameter of 20 nm and a height of 5 nm. The wetting layer is two monolayers. The measured system has been doped with sheet doping of two electrons per dot. The strain controlling layer is made of In_0.15_Ga_0.85_As and is sandwiched between two layers of GaAs each with a thickness of 1 nm. Next, there are two layers of Al_0.22_Ga_0.78_As, each with a thickness of 2 nm. The rest of the structure is made of Al_0.07_Ga_0.93_As. The dimensions of the simulated QD systems are 60 nm × 60 nm × 60 nm. The strain simulation contains around ten million atoms and the atomistic grid is as shown in Figure 2.

The band structure calculations do not need all of atoms to be included in the simulation, since bound states decay exponentially outside the quantum dot. The band structure calculations are performed using a 40 nm × 40 nm × 20 nm box surrounding the quantum dot. This box contains only 1.5 million atoms. Well-defined and well-calibrated tight-binding models are needed to enable such large-scale device simulations. Early works on tight-binding models started from analytical effective mass extractions from the usually only numerically defined model [12]. Later semi-automatic mapping methods using genetic algortithms were introduced [13] followed by DFT-based projection methods [14–15]. Each atom has 20 orbitals in the sp3d5s*_SO tight-binding basis. Strain and electronic structure simulations of such large systems are computationally demanding and require highly scalable computational codes. The code used for our simulations is the Nano Electronic MOdeling tool in version 5 (“NEMO5”) [16–22].

## Theoretical Model

### Atomistic strain model

The Harmonic Keating strain model, introduced in [6], has the elastic energy given by

**Figure 3:**
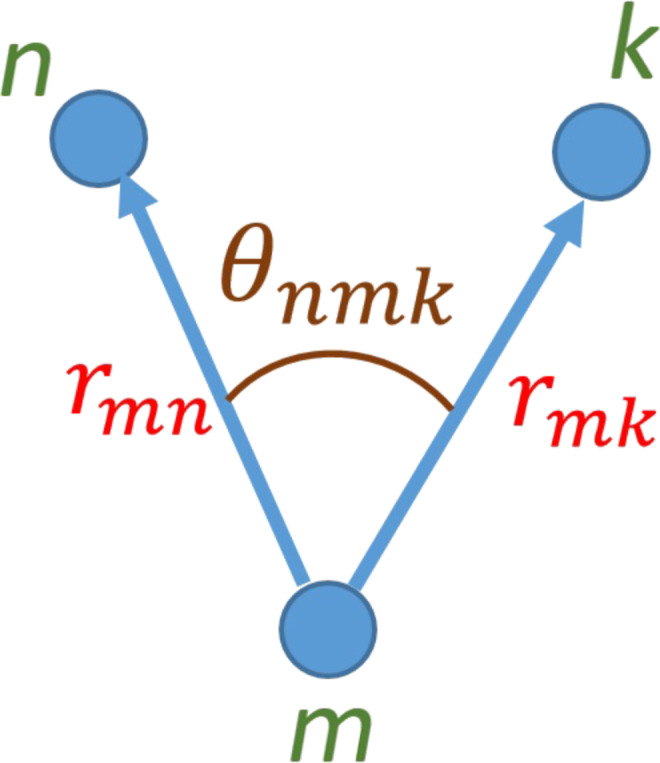
Bond lengths and bond angle for three neighboring atoms *m*, *n*, and *k*.

[1]
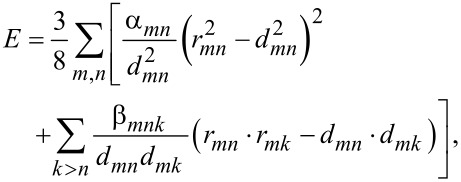


where *r_mn_* is the displacement vector from atom *m* to atom *n* for the strained crystal as shown in Figure 3, while *d_mn_* is the same vector for the unstrained crystal. The coefficient α corresponds to the force constant of the bond length distortion, the bond-stretching coefficient. While β is the bond-bending coefficient that corresponds to the force constant of the bond angle (θ) distortion. The difference between the dot products reduces to the difference in cos(θ).

The summation includes nearest neighbors only and the total energy is minimized with respect to the individual atomic positions, thus relaxing the structure. The problem with the harmonic Keating potential given by Equation 1 is that it produces a symmetric energy profile around the equilibrium interatomic distance and angle. Thus, the Keating model fails to reproduce the weakening of the strain energy with increasing bond length and underestimates the repulsive forces at close atomic separations [7–8]. The anharmonic correction of the Keating model proposed by Lazarenkova et al. [7–8] solves this problem by modifying the two parameters α and β of the Keating model and making them functions of bond length *r* and bond angle θ as given by

[2]
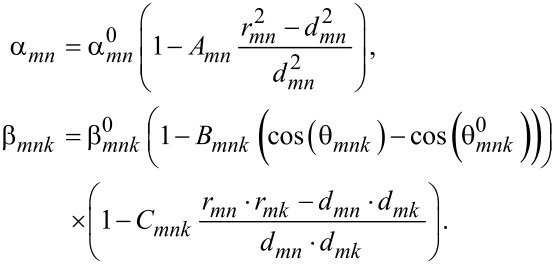


*A*, *B* and *C* are anharmonic correction coefficients. *A* and *C* describe the dependence of bond-stretching coefficient α and bond-bending coefficient β on the bond length strain, while *B* describes the dependence of bond-bending coefficient (β) on angle deformation. The anharmonic model was developed to simulate phonon dispersion and transport and the anharmonic strain parameters were optimized to reproduce the Grüneisen parameters γ*_i_* = −(*V*/ω*_i_*)(δω*_i_*/δ*V*), which are a measure of the dependence of the phonon mode frequencies on strain. Simulating the strain in quantum dots with the original anharmonic strain parameters produces inaccurate results.

**Figure 4:**
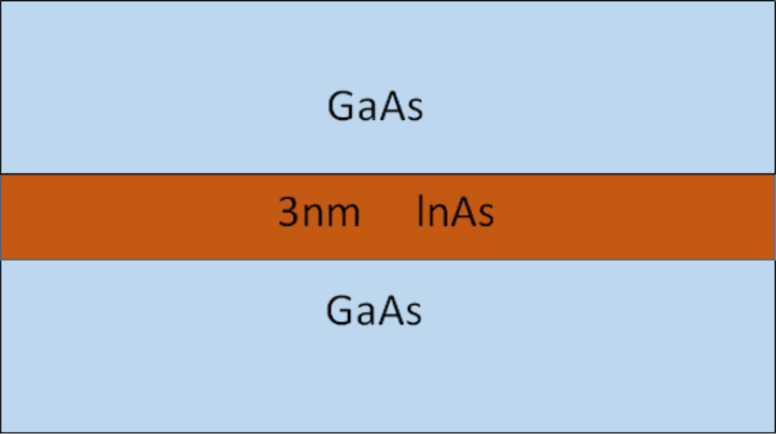
An InAs/GaAs quantum well of thickness 3 nm used for the optimization of the anharmonic strain model.

In addition, simulating strain in quantum wells with these parameters gives strain tensor components that do not match the analytical solution of the strain in quantum wells as shown in Table 1. The parameters of the model have been tuned to reproduce the biaxial strain ratio σ of InAs in order to capture the strain distribution in quantum wells and quantum dots made from InAs. The biaxial strain ratio σ of InAs is 1.053 [23]. Only the parameter α^0^ has been tuned to 19.35 Nm^−1^ while keeping the rest of the strain parameters as reported in [7]. Table 1 shows the atomistic strain calculated for InAs/GaAs quantum well, as shown in Figure 4, before and after tuning. The analytical expressions for the strain components in quantum wells are 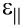 = (*a*_GaAs_ − *a*_InAs_)/*a*_InAs_ and 
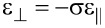
[24], where *a* is the lattice constant.

**Table 1:** Strain calculated for the InAs/GaAs quantum well. Tuning has improved the anharmonic strain results in the quantum well.

method	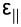	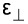

analytical	−6.68%	7.04%
anharmonic before tuning	−6.68%	8.9%
anharmonic after tuning	−6.68%	7.04%

### Electronic structure and absorption

The eigenstates of the system were calculated with a Hamiltonian constructed from semi-empirical tight-binding sp3d5s*_SO basis. The Slater–Koster tight-binding [25] parameters for InAs, GaAs and AlAs are taken from [26–27]. Boykin et al. show the effect of including strain on the tight-binding Hamiltonian [10]. These parameters are well established and previously verified with experimental measurements of quantum dots [28–31].

For the absorption coefficient α, Fermi’s golden rule has been used to calculate the absorption coefficient [32–33],

[3]
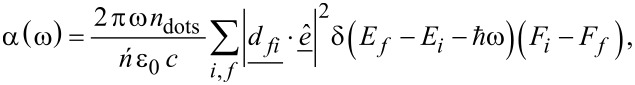


where *n*_dots_ is the number of quantum dots per unit volume, ω is the photon angular frequency, *E_i_* and *E_f_* are initial and final energies of the transition, *F_i_* and *F_f_* are occupation probability of the initial and final states, ń is the refractive index of the material, *c* is the speed of light in free space, ε_0_ is the free space permittivity, 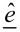 is the polarization of the incident light, and 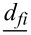 is the first-order dipole moment that is given by 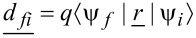, where *q* is the electron charge.

For transitions between bound states in valence and conduction bands, *F_i_* = 1, while *F_f_* depends on the energy level and doping. Normally, quantum dots are occupied by a number of electrons equal to the average number of dopants per dot [34]. This approach is reasonable for quantum dots that are far from heavily doped regions. However, it is not appropriate for quantum dots adjacent to heavily doped regions, such as contacts. In addition, to calculate optical transitions of doped quantum dots, the many-particle states of the quantum dot are evaluated using atomistic configuration interaction [35]. The method accurately captures the electron–electron interactions in electrons bound to dopant atoms in silicon. The single-particle states of the quantum dot are obtained from atomistic tight-binding calculations in NEMO5. These single-electron and hole states are used to construct many-particle Slater determinants, of all possible configurations. Using a full configuration interaction method [36], a many-particle Hamiltonian is constructed and diagonalized in the basis of Slater determinants to obtain the many-particle energies and wavefunctions.

## Results and Discussion

### Simulation versus experimental results

**Figure 5:**
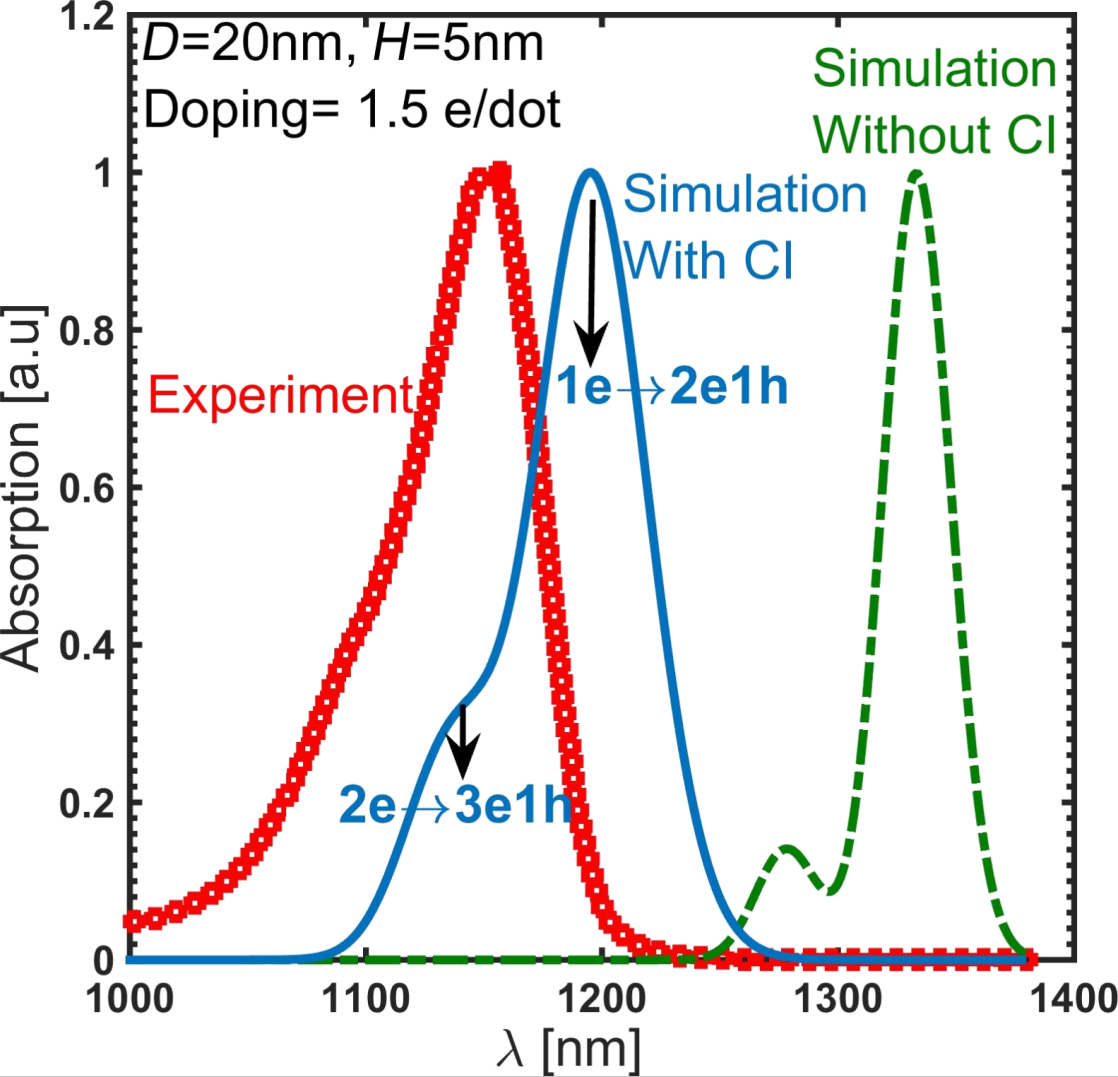
The simulated and the measured absorption spectrum of the QD system. The quantum dot is sample D from [11], which is a dome-shaped QD with a base diameter of 20 nm and height of 5 nm. The doping is 1–2 electrons per dot, which is assumed to be 1.5 here. The inclusion of many-particle configuration interaction (CI) to calculate the energy transitions significantly improves the agreement of the simulation and the experimental measurements. The higher peak corresponds to quantum dots occupying one electron (1e) transitioning to an excited state of two electrons and one hole (2e1h), while the lower peak corresponds to quantum dots occupying two electrons (2e) transitioning to an excited state of three electrons and one hole (3e1h). The error is less than 3% in calculating the absorption peak photon energy.

The model is validated with the measured absorption spectrum [11] of the QD system. Figure 5 shows the calculated and measured absorption spectrum of the device. The simulation result matches very well with the measured absorption and the error in estimating the energy of the absorption peak is less than 3%. This small error can be attributed to idealizing the quantum dot shape, ignoring the slight uncertainty in the material compositions and the variations in the quantum dot dimensions. The doping is 1.5 electrons per dot. The inclusion of many-particle configuration interaction (CI) in calculating the energy transitions significantly improves the agreement between simulations and experiment for the doped quantum dot system. The larger peak in the simulated absorption both with and without CI corresponds to quantum dots occupying one electron (1e) transitioning to an excited state of two electrons and one hole (2e1h), while the lower peak corresponds to the portion of quantum dots occupying two electrons (2e) transitioning to an excited state of three electrons and one hole (3e1h). Including the CI in the simulation results in a reduction in the absorption wavelength due to the repulsive nature of the interaction that increases the transition energy. Additional comparisons with experimental measurements are provided in the discussion of the effect of alloy mole fraction on the strain-controlling layer.

### Band structure and states

**Figure 6:**
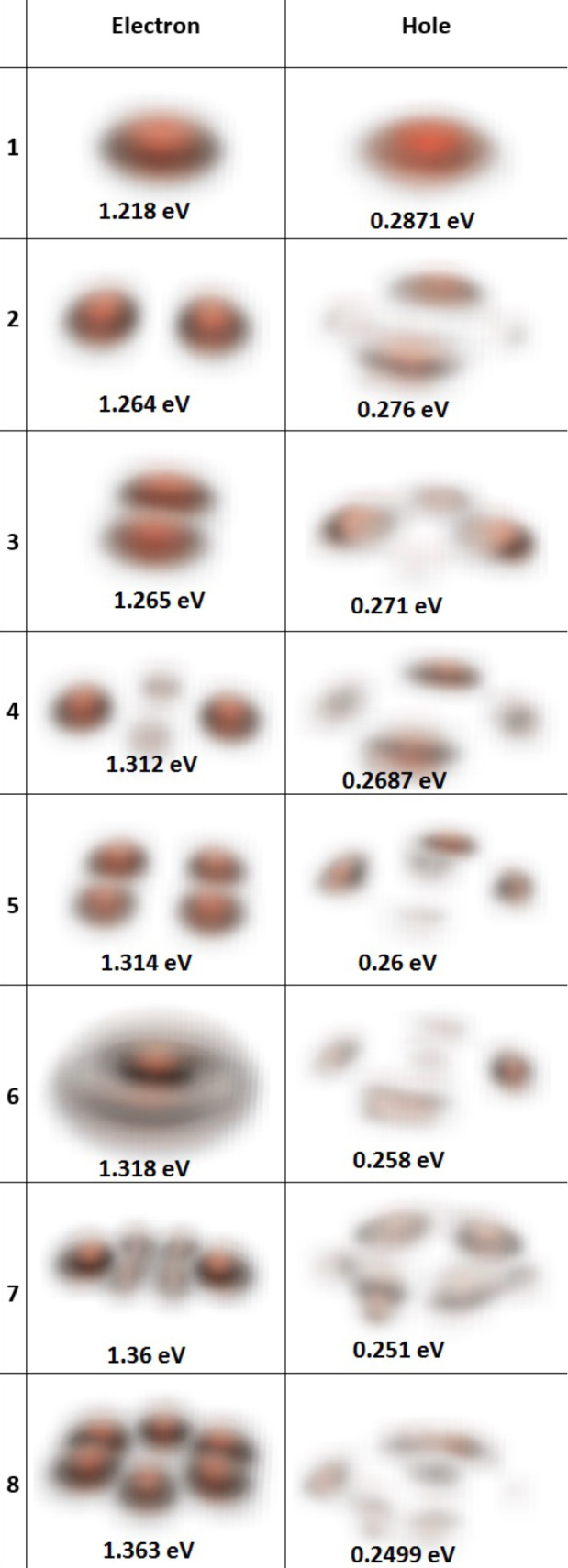
The magnitude square of the wave functions of the electron and hole states. Only the first eight electron and hole states are plotted.

Figure 6 shows the wavefunction probability density of the first eight non-degenerate states of both electrons and holes. It is worth noting that the hole ground state has an s-orbital-like shape.

**Figure 7:**
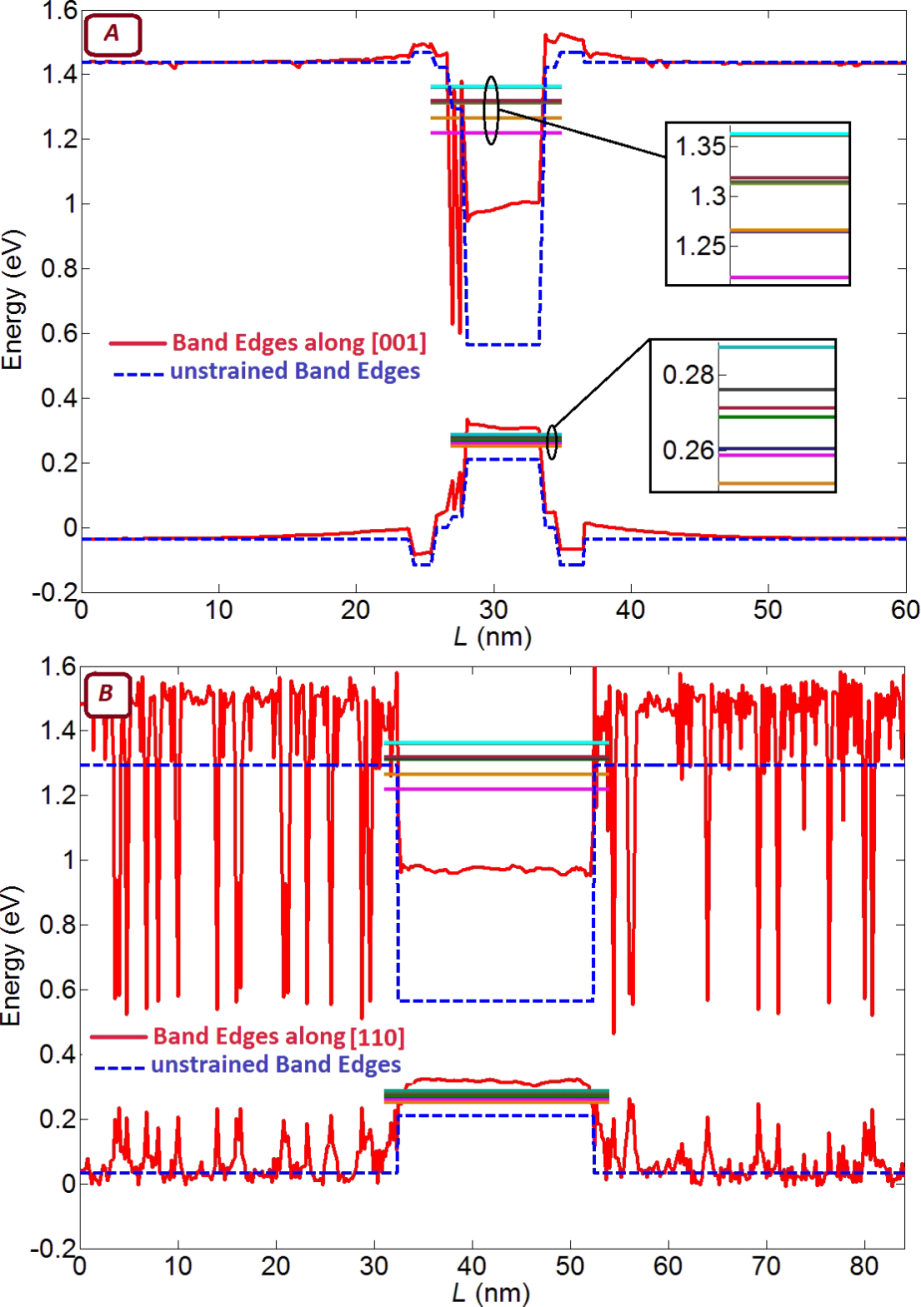
The conduction and valence band edges (solid lines) along a line through the middle of the quantum dot in the (A) [001] and (B) [110] directions. The dashed lines are the band edges of the unstrained bulk materials, drawn to show the significant effect of strain on deforming the band structure. The solid horizontal lines in the quantum dots are plotted at the energies corresponding to electron and hole confined states. The noisy red lines in (B) indicate the local band edges in the explicitly represented atomistic alloy. In an atomistic representation of an alloy one obtains an explicitly fluctuating band edge [26].

QDs have a complicated band profile since multiple effects such as geometric confinement, strain and alloy disorder, can cause major changes in the band edges of the bulk material. It is important to know where the wavefunctions of the electrons and holes are localized due to these disordered band edges, as the spatial overlap between the states determines the optical absorption spectrum. Hence, one can look at the conduction and valence band edges along arbitrary lines passing through the quantum dot. This can be done using deformation potential theory, which gives the shift of band edges due to small lattice deformations. The shift in the band edges due to lattice strain for zincblende materials is given by [37]:

[4]
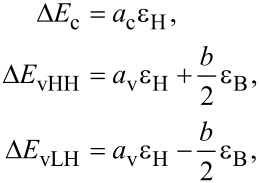


where Δ*E*_c_ is the shift in the conduction band edge, Δ*E*_vHH_ and Δ*E*_vLH_ are the shifts in the heavy and light hole band edges, respectively. *a*_c_, *a*_v_, and *b* are the deformation potential coefficients of the material. In these simulations, the parameters recommended for III–V materials by [38] are used. ε_H_ and ε_B_ are the hydrostatic and biaxial strain components, which are linear combinations of the atomistic strain components: ε_H_ = ε*_xx_* + ε*_yy_* + ε*_zz_* and ε_B_ = ε*_xx_* + ε*_yy_*− 2ε*_zz_* [37], where *z* is the growth direction. Figure 7 shows the band edges along two lines through the middle of the quantum dot along the [001] and [110] directions. The unstrained band edges show a significant effect of strain on the band edges.

### Effect of quantum dot dimensions

**Figure 8:**
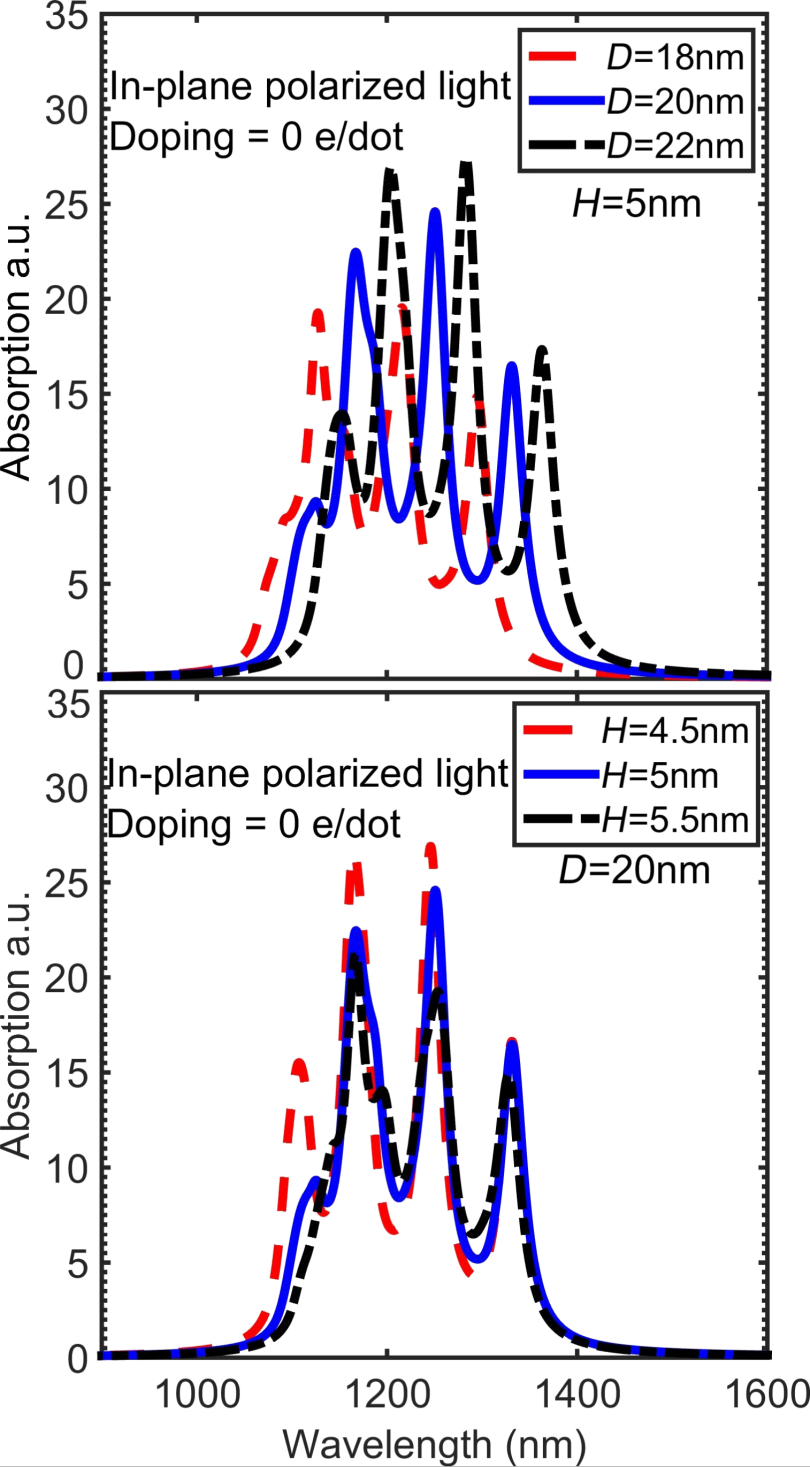
The in-plane polarized absorption spectrum calculated for (A) different diameters and (B) different heights of the quantum dot. Increasing the dot diameter results in a shift of the peaks towards longer wavelengths, while increasing the dot height does not have a significant effect on the wavelength.

Figure 8 shows the effect of variations in quantum dot diameter and height on the in-plane polarized absorption spectrum. An increasing dot diameter results in a red-shift of the absorption peaks, while increasing the dot height does not have a significant effect on the absorption wavelength. In contrast to the simple particle-in-a-box problem, which predicts a stronger sensitivity to the smaller dimension (the height), our simulations show that absorption wavelength is much more sensitive to changing the dot diameter than to changing the height.

The effects of changing dimensions on the energy transition Δ*E* between the hole and electron ground states can be understood with a simple analytical model. This transition has two contributions: strain and confinement. The strain shifts the band edges and affects the energy gap *E*_g_, while the confinement increases the minimum allowed energy of electron *E*_elec_ and hole *E*_hole_ with respect to the band edges. Let *E*_box_ = *E*_elec_ + *E*_hole_, then the transition energy *E* is

[5]
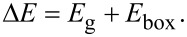


Due to the sign of the deformation potential and strain, the valence band edge inside the quantum dot is of a heavy hole, from Equation 4

[6]
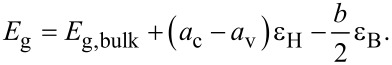


**Figure 9:**
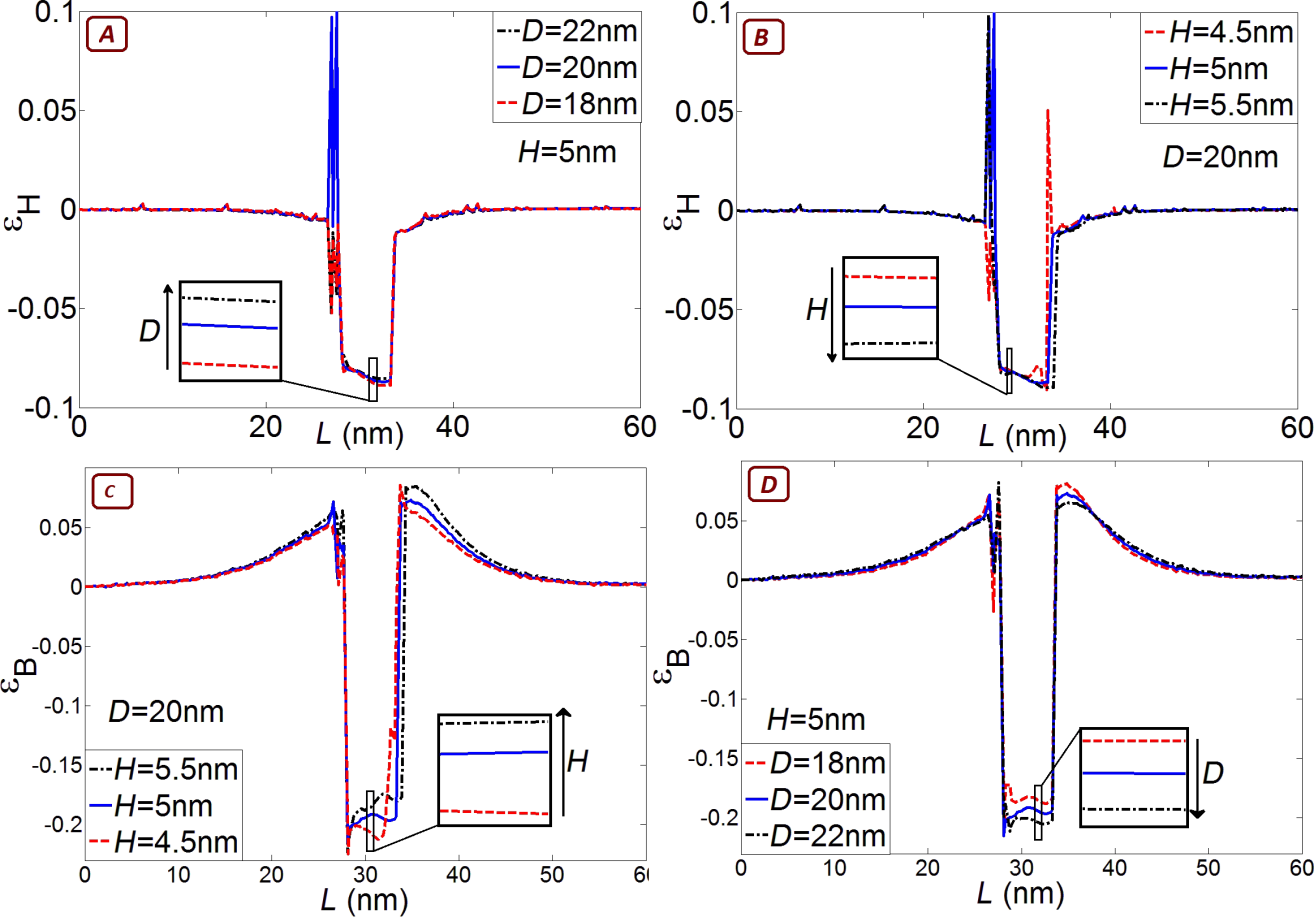
Hydrostatic ε_H_ and biaxial ε_B_ strain with different dimensions along a line through the middle of the quantum dot in the [001] direction. (A) ε_H_ as a function of different dot diameters; (B) ε_H_ as a function of different dot heights; (C) is ε_B_ as a function of different dot diameters; (D) ε_B_ as a function of different dot heights. The magnitude of the biaxial strain increases with increasing diameter and decreases with increasing height, while the hydrostatic strain evolves in the opposite direction.

Figure 9 shows the effect of changing diameter and height on the hydrostatic and biaxial strain. The magnitude of the biaxial strain increases with increasing diameter and decreases with increasing height, while the magnitude of the hydrostatic strain changes slightly in the direction opposite to the biaxial strain. Increasing the height is equivalent to the decreasing diameter in terms of changing the strain in the quantum dot since it depends almost entirely on the aspect ratio not on the individual dimensions [39]. Increasing the diameter reduces the energy gap, which further reduces the optical transition energy, while increasing the height increases the energy gap, which works against the reduction in confinement energy. In the case of varying height, this compensation results in almost the same optical transition energy. Although the variations in the hydrostatic strain are smaller than variations in the biaxial strain, as shown in Figure 9, the hydrostatic strain variations cannot be neglected. This is due to the stronger weight of deformation potential for hydrostatic strain. For example, *a*_c_ − *a*_v_ = -6 eV is six times higher than *b*/2 = −1 eV for InAs. Also, changing one of the dimensions either increases or decreases the hydrostatic strain, and it has the opposite effect on biaxial strain (decreases or increases), but the hydrostatic and biaxial strain work together in the same direction on the energy gap since they have opposite signs in Equation 6.

To get an expression for *E*_box_, the dome-shaped quantum dot is approximated to be a disc of cylinder radius *R* and height *H*. One can easily obtain *E*_box_ by solving an effective-mass Hamiltonian in the cylindrical coordinates,

[7]
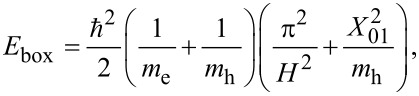


where *m*_e_, *m*_h_ are the effective masses of electron and the heavy hole, and *X*_01_ = 2.405 is the Bessel function of the first kind with order zero. Note that effective masses for the electron and heavy hole under strain are different from that in the bulk, and the effective masses of InAs are *m*_e_ = 0.1*m*_0_ and *m*_h_ = 0.48*m*_0_ [26].

For a quantum dot of *D* = 20 nm and *H* = 5 nm, the sensitivity of the confinement energy to the dot radius is


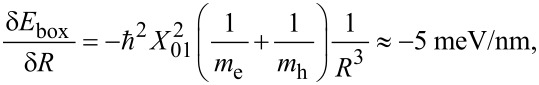


and the sensitivity of the energy gap to the dot radius is


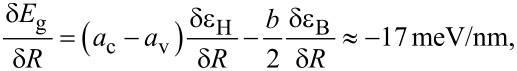


which give a total sensitivity of the optical transition (δΔ*E*)/δ*R* ≈ −22 meV/nm.

Similarly for variations in height,


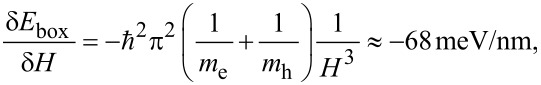



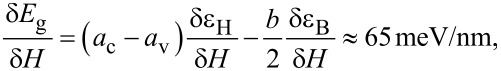


which give (δΔ*E*)/δ*H* ≈ −3 meV/nm. Increasing the dot radius causes both contributions to reduce the transition energy. Increasing the dot height causes both contributions to work against each other, which reduces the sensitivity of the transition energy to dot height.

### Strain controlling layer

**Figure 10:**
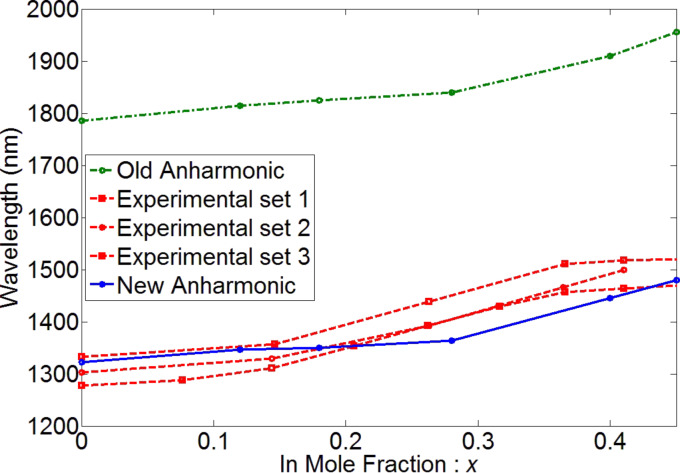
Experimental and simulation results of the optical transition of the QD system reported in [9]. Increasing the In mole fraction increases the transition wavelength. The optimization of the anharmonic strain model has greatly improved the simulation results.

**Figure 11:**
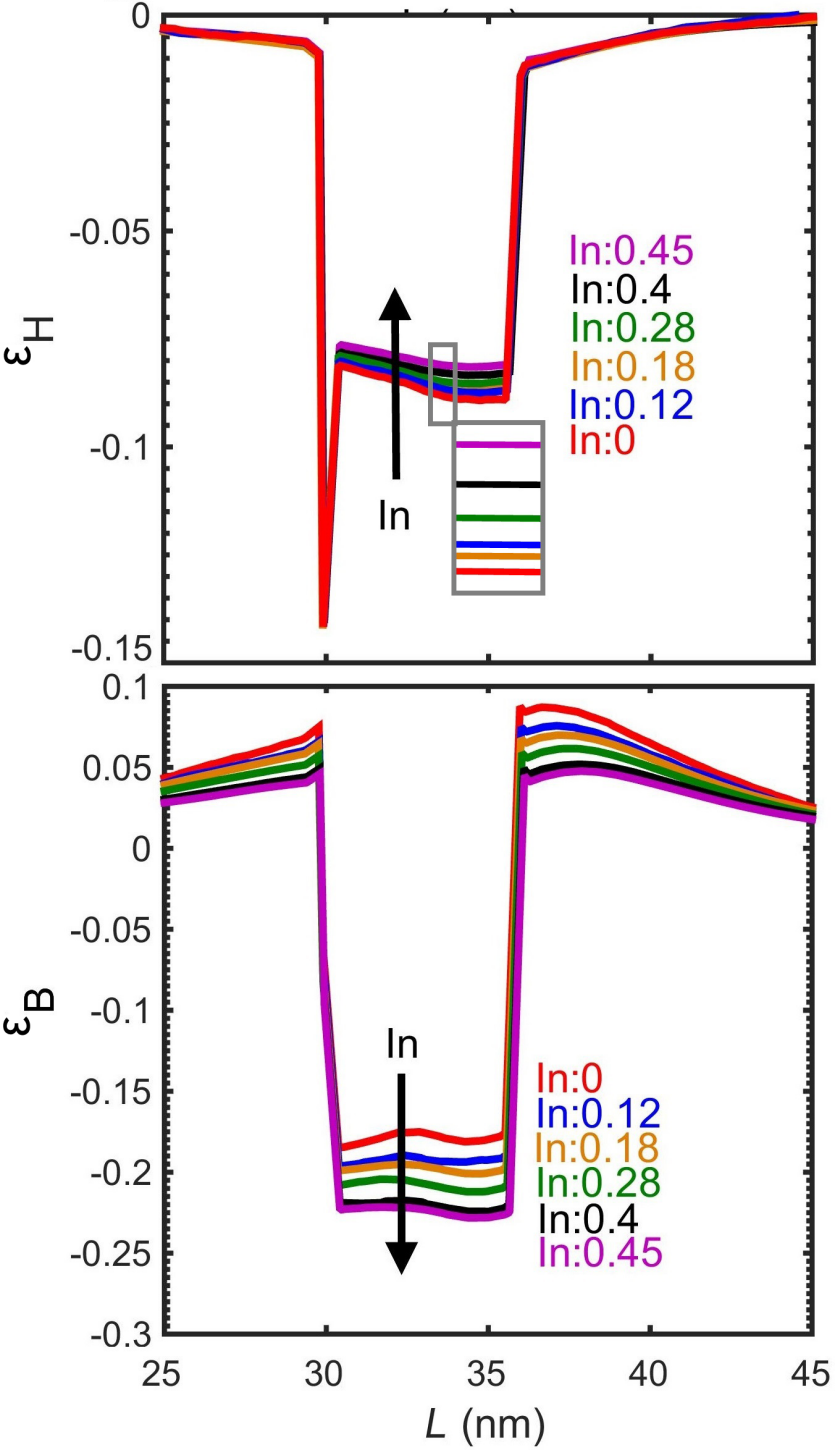
Hydrostatic and biaxial strain with different mole fractions of In along a line passing through the middle of the quantum dot in the [001] direction.

Changing the mole fraction of In in the InGaAs strain controlling layer (capping layer) is a convenient way to tune the absorption peak. The effect of mole fraction has been studied on a slightly different system, reported in [9], which helps us further validate the results of the simulations. The system reported in [9] is almost the same as in [11] except for two differences: (i) it is not doped and, (ii) it uses GaAs instead of AlGaAs. Figure 10 shows the experimental and simulation results of the optical transitions of the QD systems reported in [9]. Unlike the earlier discussed experiments, quantum dots in [9] are undoped and have been measured at various strain controlling layer compositions. The optimization of the anharmonic strain model greatly improves the simulation results. Increasing the mole fraction of In increases the transition wavelength. This is further explained by examining the effect of changing the mole fraction on hydrostatic and biaxial strain and the band edges. Figure 11 shows the hydrostatic and biaxial strain along a lines passing through the middle of the quantum dot in the [001] direction for different mole fractions of In. As shown in these figures, the hydrostatic and biaxial strain change with the In mole fraction in the same way they change with diameter. That is, increasing the In mole fraction results in an increase in the magnitude of the biaxial strain and a decrease in the magnitude of the hydrostatic strain. This leads to a lower energy gap and larger absorption wavelength, as shown in Figure 10.

## Conclusion

In this paper, a detailed theoretical study of the optical absorption and strain behavior in self-assembled quantum dots has been presented. Self-assembled quantum dots are highly strained heterostructures and a rigorous atomistic strain model is needed to accurately calculate the electronic states in the system. In addition, many-particle configuration interaction has been accounted for, to properly simulate doped quantum dots. The models accurately describe the complex coupled underlying physics. This improvement is shown by the closer agreement with experimental data. The simulations reproduce the experimental results with an error below 3%. The model was implemented in NEMO5 and used to simulate characteristics of an InAs/GaAs/AlAs quantum dot systems. Increasing the dot diameter results in a shift of the absorption peaks towards longer wavelengths, while increasing the dot height does not have a significant effect on wavelength. When the diameter is changed, the band gap and confinement energies work with each other, whereas when the height is changed, the band gap and confinement energies work against each other. Increasing the mole fraction if In in the strain controlling layer works in the same way as increasing the dot diameter and changes the strain leading to longer absorption wavelengths.

In conclusion, the method presented here provides a way to incorporate the inhomogeneous environment of QDs in simulations by taking into account device geometry and quantum confinement, alloy disorder, electrostatics, many-particle interactions, and spatially varying strain distribution. Such details are needed to interpret and guide experimental measurements and device design with quantitative accuracy.
